# Placement of an elastic, biohybrid patch in a model of right heart failure with pulmonary artery banding

**DOI:** 10.3389/fbioe.2024.1485740

**Published:** 2025-01-20

**Authors:** Yasunari Hayashi, Seungil Kim, Taro Fujii, Drake Dalton Pedersen, Takahiro Ozeki, Hongbin Jiang, Antonio D’Amore, William R. Wagner

**Affiliations:** ^1^ McGowan Institute for Regenerative Medicine, University of Pittsburgh, Pittsburgh, PA, United States; ^2^ Department of Surgery, University of Pittsburgh, Pittsburgh, PA, United States; ^3^ Department of Bioengineering, University of Pittsburgh, Pittsburgh, PA, United States; ^4^ Department of Agricultural and Biological Engineering, Mississippi State University, Mississippi State, MS, United States; ^5^ Fondazione RiMED, Palermo, Italy; ^6^ Medicina di Precisione in Area Medica, Chirurgica e Critica, University of Palermo, Palermo, Italy

**Keywords:** right heart failure, pulmonary artery banding, right ventricular hypertrophy, cardiac extracellular matrix, cardiac patch

## Abstract

**Introduction:**

In a model of right heart failure secondary to pulmonary artery banding (PAB), a mechanical approach using an elastic, biodegradable epicardial patch with integrated extracellular matrix digest was evaluated for its potential to inhibit disease progression.

**Methods:**

Adult male syngeneic Lewis rats aged 6–7 weeks old were used. Biohybrid cardiac patches were generated by co-processing biodegradable poly(ester carbonate urethane) urea (PECUU) and a digest of the porcine cardiac extracellular matrix. Three weeks after PAB, the cardiac patch was attached to the epicardium of the right ventricle (RV). Cardiac function was evaluated using echocardiography and catheterization for 9 weeks after PAB, comparing the patch (n = 7) and sham (n = 10) groups.

**Results:**

Nine weeks after PAB, the RV wall was thickened, the RV cavity was enlarged with a reduced left ventricular cavity, and RV wall interstitial fibrosis was increased. However, these effects were diminished in the patch group. Left ventricular ejection fraction in the patch group was higher than in the sham group (*p* < 0.001), right end-systolic pressure was lower (*p* = 0.045), and tricuspid annular plane systolic excursion improved in the patch group (*p* = 0.007). In addition, von Willebrand factor expression was significantly greater in the patch group (*p* = 0.007).

**Conclusions:**

The placement of a degradable, biohybrid patch onto the RV in a right ventricular failure model with fixed afterload improved myocardial output, moderated pressure stress, and was associated with reduced right ventricular fibrosis.

## 1 Introduction

Right ventricular function plays an important role in the prognosis of congenital heart disease, pulmonary hypertension, dilated cardiomyopathy, and left heart failure. This is because prolonged exposure to right ventricular overload results in right ventricular hypertrophy and a gradual decline in diastolic function (Meyer et al., 2010). Previous experimental studies have most commonly evaluated right ventricular function in models of left heart ischemia or pulmonary hypertension. Although these models provide insights into non-fixed-afterload conditions, pulmonary artery banding (PAB) with clips provides a highly reproducible fixed-afterload model, which allows for the investigation of direct cardiac interventions regardless of pulmonary vascular resistance (Yogeswaran et al., 2023). Pulmonary artery banding is an established procedure used in pediatric cardiac surgery to limit blood flow to the pulmonary artery in complex congenital conditions.

In previous efforts to address left ventricular remodeling in ischemic cardiomyopathy, we developed a mechanical approach involving the placement of an elastic, biodegradable patch on the ischemic wall to redirect the remodeling pathway and preserve left ventricular output (D’Amore et al., 2016). This approach, with temporary mechanical support, was hypothesized to be valuable in the treatment of the dilated right ventricle and the preservation of cardiac function in right heart failure. A rat model for right heart failure with pulmonary artery banding was used, with epicardial placement of an elastic, biohybrid patch to assess the potential of this approach to interrupt disease progression.

## 2 Materials and methods

### 2.1 Generation of elastic, biohybrid patches

**FIGURE 1:**
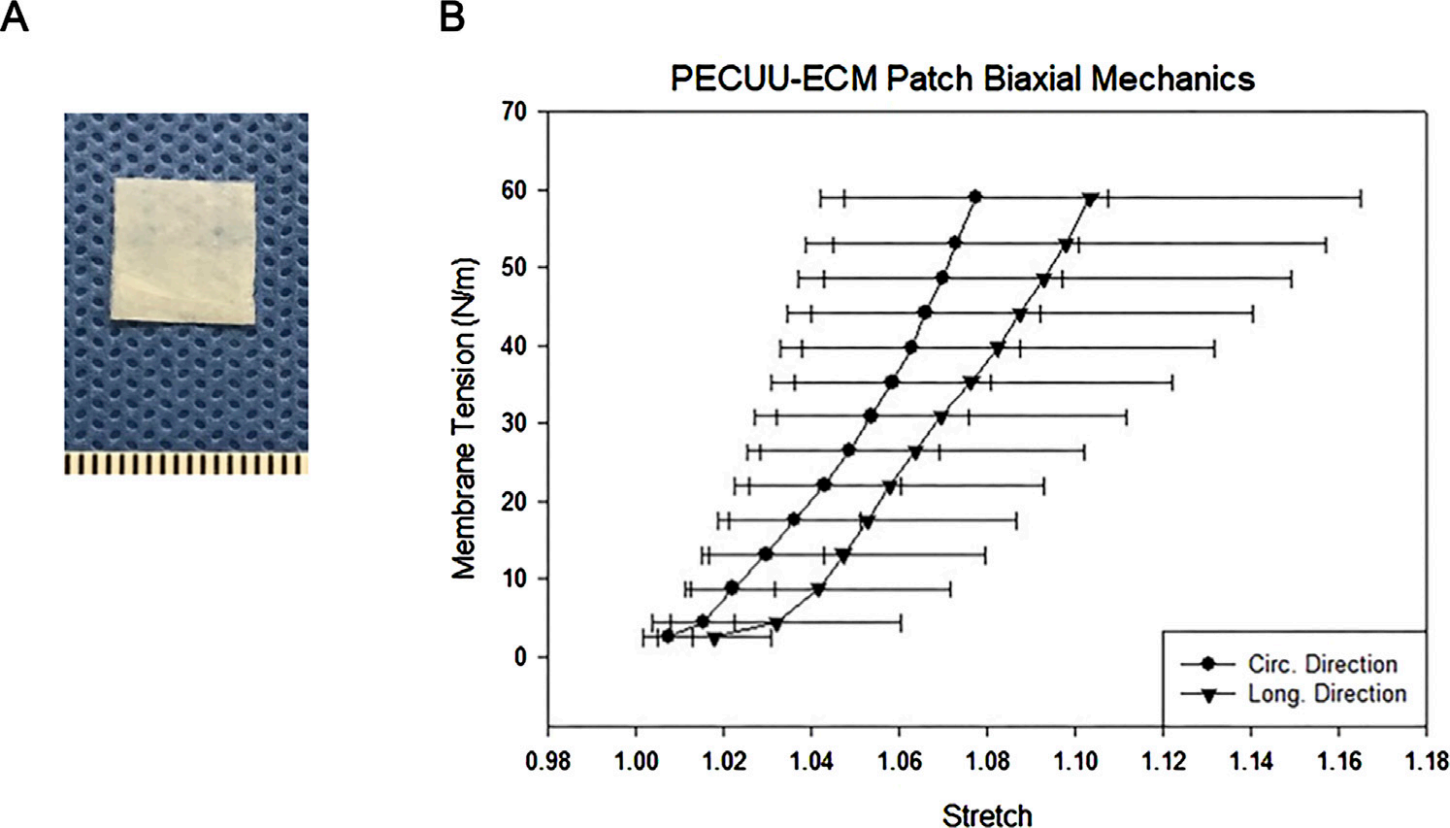
**(A)** Biohybrid patch (mm scale). **(B)** Biaxial tensile properties of the biohybrid patch in the circumferential and longitudinal directions.

Biodegradable, elastic cardiac patches incorporating the cardiac extracellular matrix (ECM) were generated as described previously (D’Amore et al., 2016). In brief, intact porcine hearts (Thomas’ Meat Market, Saxonburg, PA, United States) were decellularized through retrograde aortic perfusion, and cardiac ECM hydrogel was generated as described previously (Remlinger, Wearden, and Gilbert, 2012). Cardiac patches were made by electrospinning synthesized poly(ester carbonate urethane) urea (PECUU) (Hashizume et al., 2013) while concurrently electrospraying a saline solution first and then a cardiac ECM hydrogel to create a biohybrid sheet of microfibrous polymer with a distinct region containing ECM hydrogel between the fibers. Square patches (8 mm/side) large enough to cover the dilated rat right ventricular wall were cut from this sheet (Figure 1).

### 2.2 Animal models

**FIGURE 2:**
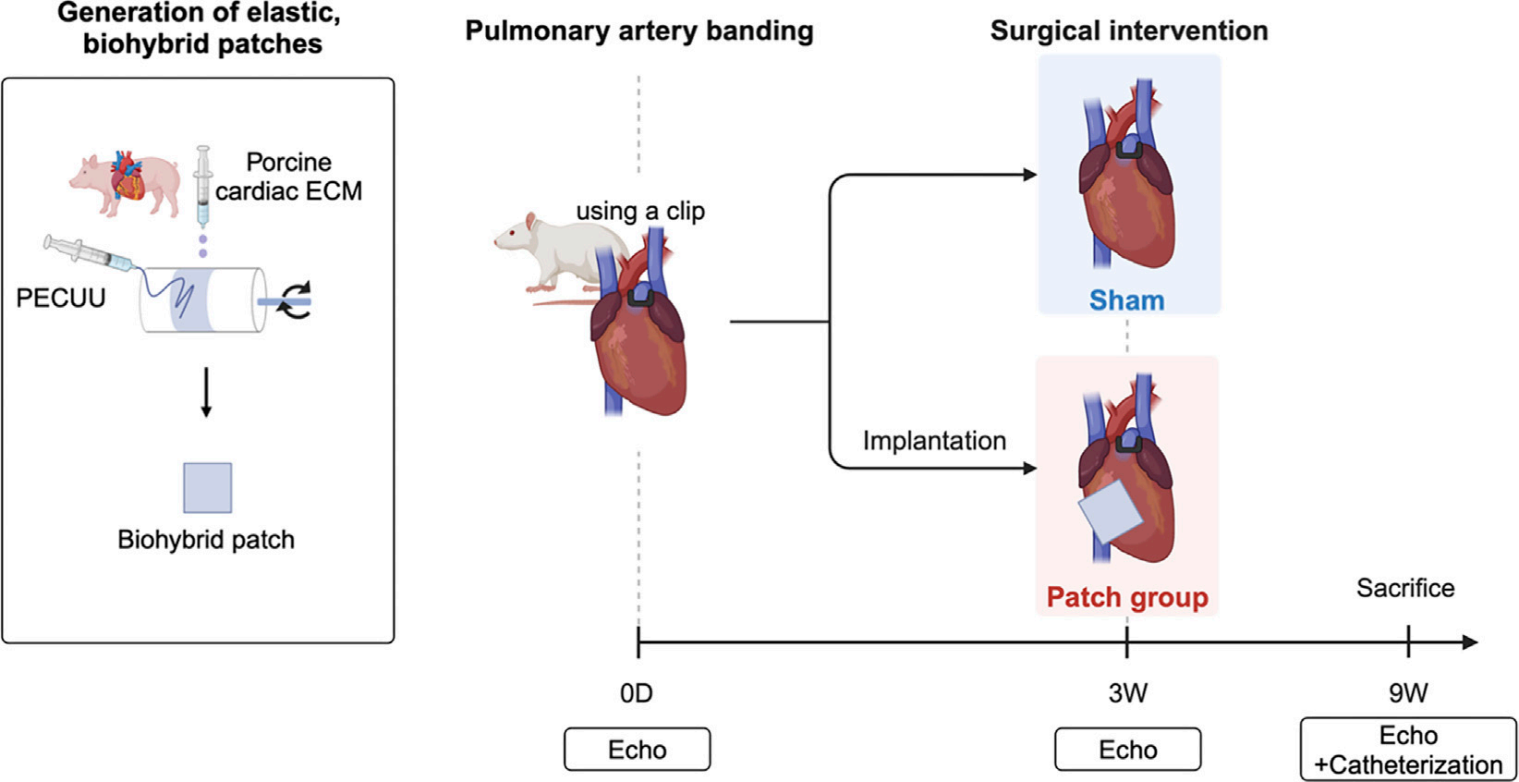
Overall experimental approach with defined groups and follow-up examinations after pulmonary artery banding. Created in BioRender. Hayashi, Y. http://BioRender.com/q12b428.

Adult male syngeneic Lewis rats aged 6–7 weeks old (ENVIGO, Indianapolis, IN, United States of America) were used for this study. Rats were divided into two groups: a patch group (n = 7), where the biohybrid (PECUU-cardiac ECM hydrogel) patch was applied 3 weeks after PAB, and a sham group (n = 10), who underwent a sham procedure 3 weeks post-PAB (Figure 2). A control group (n = 4) with no PAB or patching was used for morphometric comparisons. During the study, nine rats died before patch or sham surgery, and these animals were not included in the group numbers listed above. Specifically, seven rats were euthanized for severe right heart failure with tachypnea and immobility following the protocol several days after PAB, which our veterinarian also pointed out, and two rats died during the PAB surgery. The research protocol followed the National Institutes of Health guidelines for animal care and was approved by the Institutional Animal Care and Use Committee of the University of Pittsburgh (#20087552).

### 2.3 Right heart failure creation by pulmonary artery banding and patch placement

Right heart failure was created by stenosis generation in the main pulmonary artery trunk, as previously published by Hirata et al. (2015) and Andersen et al. (2018). Briefly, rats were anesthetized with isoflurane (2.5% for induction and 1.25%–1.5% for maintenance with 100% oxygen) and intubated. Mechanical ventilation was provided with a rodent volume-controlled mechanical ventilator (683 Ventilator, Harvard Apparatus, Holliston, MA, United States) at a tidal volume of 3 mL and 80 breaths/min. Rats were placed in the supine position on a warming blanket (37°C), and the chest was shaved and prepared with povidone–iodine solution. The procedures were performed in a sterile environment. Before the skin incision, 5 mg/kg lidocaine hydrochloride was administered intramuscularly for pain, along with cefazolin for antibiotic prophylaxis.

**FIGURE 3:**
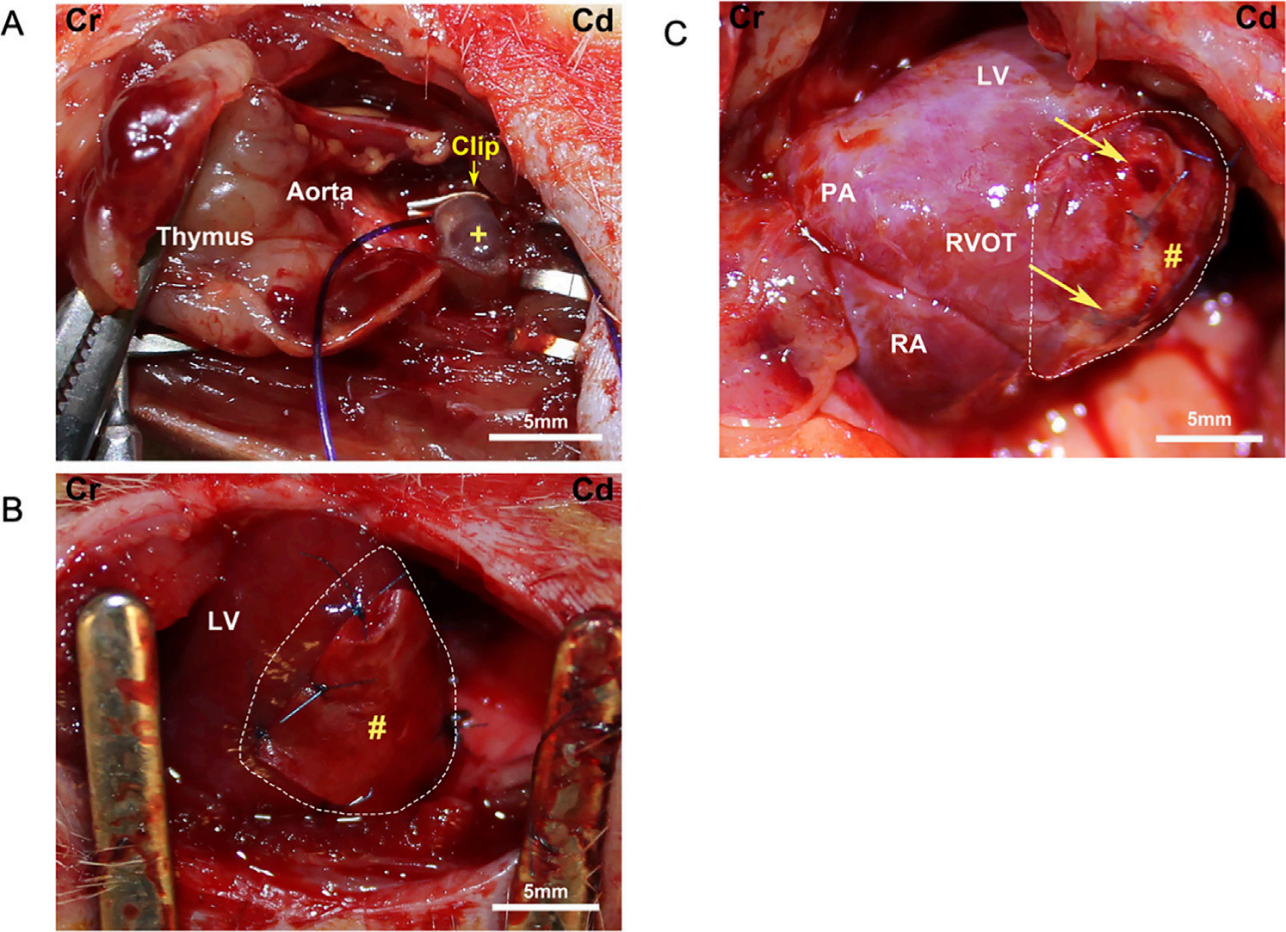
Surgical field images. Cr, cranial; Cd, caudal. **(A)** Pulmonary artery banding schema. +, pulmonary artery. **(B)** Placement of a biohybrid patch on the right ventricular surface (enclosed by dotted line). LV, left ventricle; #, patch. **(C)** Macroscopic image of a patch group heart at the end of the experiment. Arrows point to the corners of the remnant patch (#) on the right ventricular surface (enclosed by dotted line). PA, pulmonary artery; LV, left ventricle; RA, right atrium; RVOT, right ventricular outflow tract.

The rat heart was exposed through a third left thoracotomy. After identifying the main pulmonary trunk, a half-closed tantalum clip (Ethicon Inc., Bridgewater, NJ, United States) was compressed around the main trunk (Figure 3A). The clip inner diameter was adjusted to 0.6 mm, and the inner area was 1.1 mm^2^. The incision was closed in layers with 4–0 polypropylene continuous sutures. The animals were allowed to recover from anesthesia and returned to their cages after extubating. Cefazolin (20 mg/kg) and buprenorphine hydrochloride (0.1 mg/kg) were injected twice a day for 3 days post-procedure to prevent surgical site infection and manage pain.

Patch implantation procedures were performed 3 weeks after PAB. Right heart failure was confirmed by evaluating the right ventricular dilatation and distension of the ventricular septum with echocardiography. All rats were anesthetized, and the heart was exposed with the same skin incision through the fourth thoracotomy. After exposing the heart, the patch was attached to the right ventricular wall with 7–0 polypropylene knotted sutures (Figure 3B). The patches were not pretensioned during the procedure and were placed in a random orientation (circumferential versus longitudinal, aligned with a long axis) on the heart. In the sham group, the chest of the PAB animals was opened at 3 weeks, but no patch was placed. The incision was closed as described above.

### 2.4 Echocardiography

Cardiac functional parameters were recorded with echocardiography before PAB placement surgery, 3 weeks after PAB (and right before patch application), and 6 weeks after patch application (9 weeks overall). Echocardiographic analysis was performed in a blinded manner with respect to the treatment group. Rats were anesthetized with 1.5%–2.0% isoflurane inhalation with 100% oxygen. Transthoracic echocardiography was performed using the Acuson Sequoia C256 System with a 13-MHz linear ultrasonic transducer (15L8; Acuson Corporation, Mountain View, CA, United States) in a phased array format. The right ventricular (RV) morphology was evaluated by the RV outflow tract (RVOT), RV end-diastolic area (RVEDA), and RV end-systolic area (RVESA). To assess RV function, the tricuspid annular plane systolic excursion (TAPSE) of the lateral portion of the tricuspid annular plane was measured by the base-to-apex shortening during systole. Left ventricular (LV) parameters recorded were the end-diastolic area (LVEDA), end-systolic area (LVESA), end-diastolic dimension (LVDd), and end-systolic dimension (LVDs), as obtained from the short-axis view at the papillary muscle level. The fractional area change (%FAC) and fractional shortening (%FS) were calculated as %FAC = (LVEDA-LVESA)/EDA × 100% and %FS = (LVDd-LVDs)/LVDd x 100%, respectively. Ventricular volume (V) was estimated using the formula of Teichholz to calculate LV end-diastolic volume (LVEDV) and end-systolic volume (LVESV) as follows: V = 7.0/(2.4 + D) × D^³^, where D is the LV diameter measured by M-mode echocardiography. The LV ejection fraction (LVEF) was calculated as LVEF = (LVEDV–LVESV)/LVEDV x 100% (Lang et al., 2015).

### 2.5 Catheterization

Cardiac catheterization was performed to evaluate the cardiac pressure 6 weeks after patching since RV function depends on the preload and afterload. After echocardiography, the heart was exposed via median sternotomy to perform catheterization under general anesthesia (Figure 3C). The 2 F micromanometer-tipped catheter (Model SPR-838 Millar Instruments, Houston, TX, United States) was inserted via the right ventricular apex and advanced into the right ventricle to obtain RV and pulmonary artery pressure. Pressure–volume loop data were obtained during inferior vena cava occlusion. In addition, the catheter was inserted via the left ventricular apex into the left ventricle to measure the left ventricular pressure (Ma et al., 2016). After catheterization, the animals were euthanized by direct injection of 10 meq/kg KCl into the heart chamber under deep anesthesia with 5.0% isoflurane.

### 2.6 Histology and immunohistochemistry

Cross-sections of the heart transversed at the center of the left ventricle were fixed with 10% buffered formalin and embedded in paraffin. Serial paraffin-embedded sections (5 um thick) were deparaffinized in xylene; dehydrated in graded ethanol mixtures; and stained with hematoxylin and eosin, Masson’s trichrome, and picrosirius red (PSR). Paraffin-embedded sections were blocked with the staining buffer for 1 hour (10% goat serum with 1% bovine serum albumin in phosphate-buffered saline) and incubated with rabbit polyclonal anti-von Willebrand factor antibody (vWF; 1:200, ab6994, Abcam, Cambridge, MA, United States), anti-αSMA (ab5694, Abcam, Cambridge, MA, United States), and anti-CD68 (ab125212, Abcam, Cambridge, MA, United States). Nuclei were stained with 4′,6-diamidino-2-phenylindole dihydrochloride (DAPI, ab104139, Abcam, Cambridge, MA, United States). Fluorescein-labeled wheat germ agglutinin (WGA, 1:100, FL-1021–5, Vector Laboratories, Burlingame, CA, United States) and fluorescein-labeled *Griffonia simplicifolia* lectin I isolectin B4 (Isolectin, 1:100, FL-1201–0.5, Vector Laboratories, Burlingame, CA, United States) were used to identify myocyte boundaries and endothelial cells, respectively (Sun et al., 2018; Trac et al., 2019). Multispectral epifluorescent images were acquired using a Nikon Eclipse 6600 Microscope (Nikon Corporation, Tokyo, Japan) with spectral unmixing to remove autofluorescence using Nuance 3.0.2 software (Caliper Life Science Inc., Hopkinton, MA, United States). Right ventricular wall thickness was calculated as right ventricle area/[(epicardial circumference + endocardial circumference)/2].

### 2.7 Statistical analyses

Continuous values were expressed as the mean with a standard error if they were normally distributed and were compared using ANOVA with a *post hoc* Tukey test for three groups or a *t*-test for two groups. If the data were not normally distributed, they were expressed as medians with interquartile range, and we used the Kruskal–Wallis test for three groups or the Mann–Whitney *U* test for two groups. STATA software version 15.1 (StataCorp LLC, College Station, TX, United States) was used for all statistical analyses.

## 3 Results

### 3.1 Material characteristics

The tensile properties of the biohybrid patch measured by biaxial mechanical testing are shown in Figure 1B. The patch had a peak tensile strength of 60 N/m, with a peak tensile strain of 110 ± 6%. There was no statistical difference between the circumferential and longitudinal tensile behavior.

### 3.2 Cardiac hypertrophy and fibrosis

**FIGURE 4:**
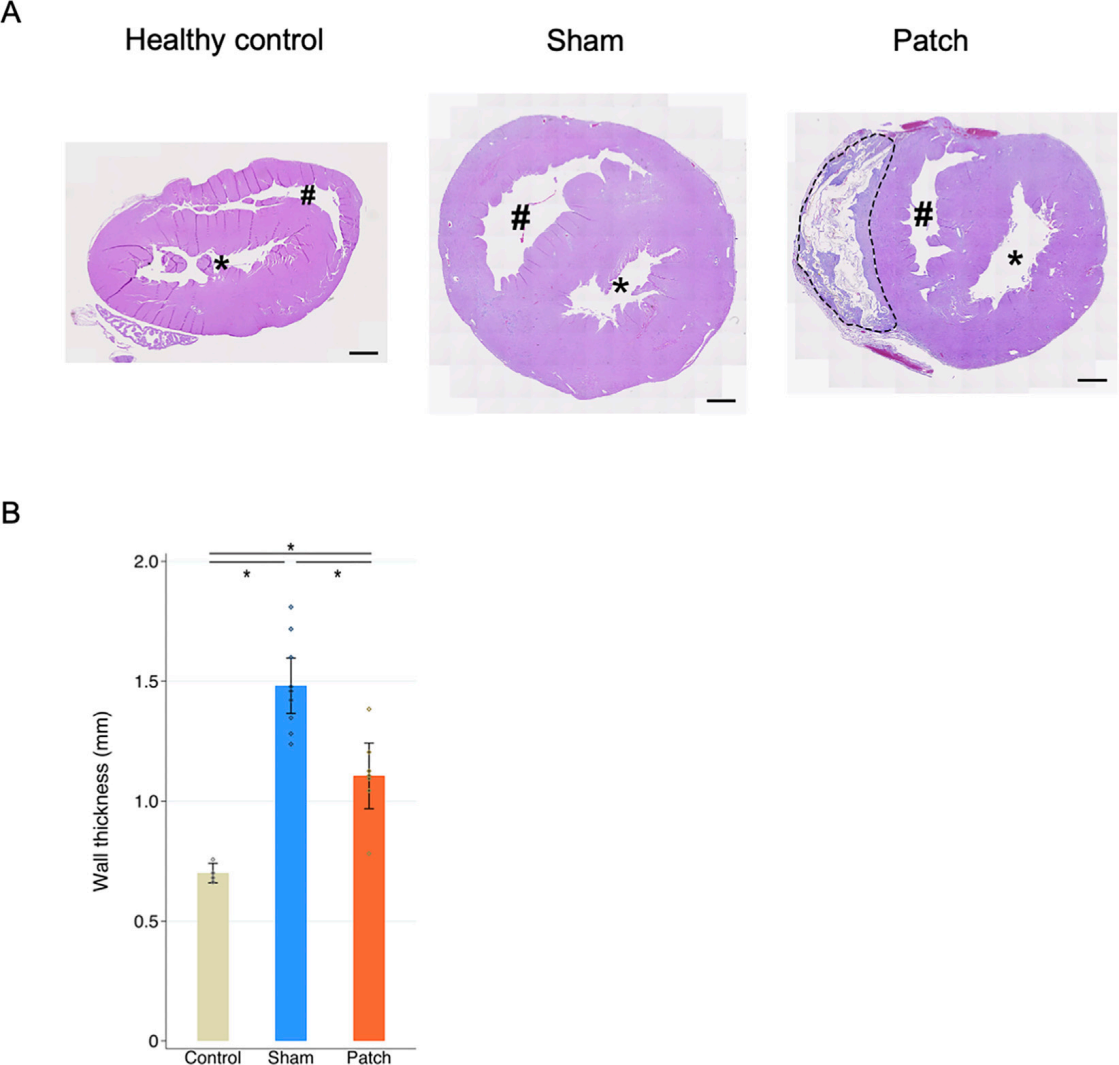
**(A)** Hematoxylin and eosin staining. Heart cross-sections illustrating morphologic changes in rats’ hearts 9 weeks after sham and pulmonary artery banding procedures relative to healthy control animals. **(B)** Right ventricle wall thickness. The sham group had significantly thicker right ventricular wall than the other two groups. *p < 0.05. Scale bars = 1 mm #, right ventricular cavity; *, left ventricular cavity. The patch remnant with tissue ingrowth is enclosed by the dotted line.

RV wall thickness 9 weeks after PAB, as assessed by hematoxylin and eosin staining of the transected whole heart, revealed a thickened RV wall (Figure 4A). The sham group had a significantly thicker RV wall than the others (healthy, 0.70 ± 0.02; sham, 1.48 ± 0.06; and patch 1.11 ± 0.07, *p* < 0.001, Figure 4B).

**FIGURE 5:**
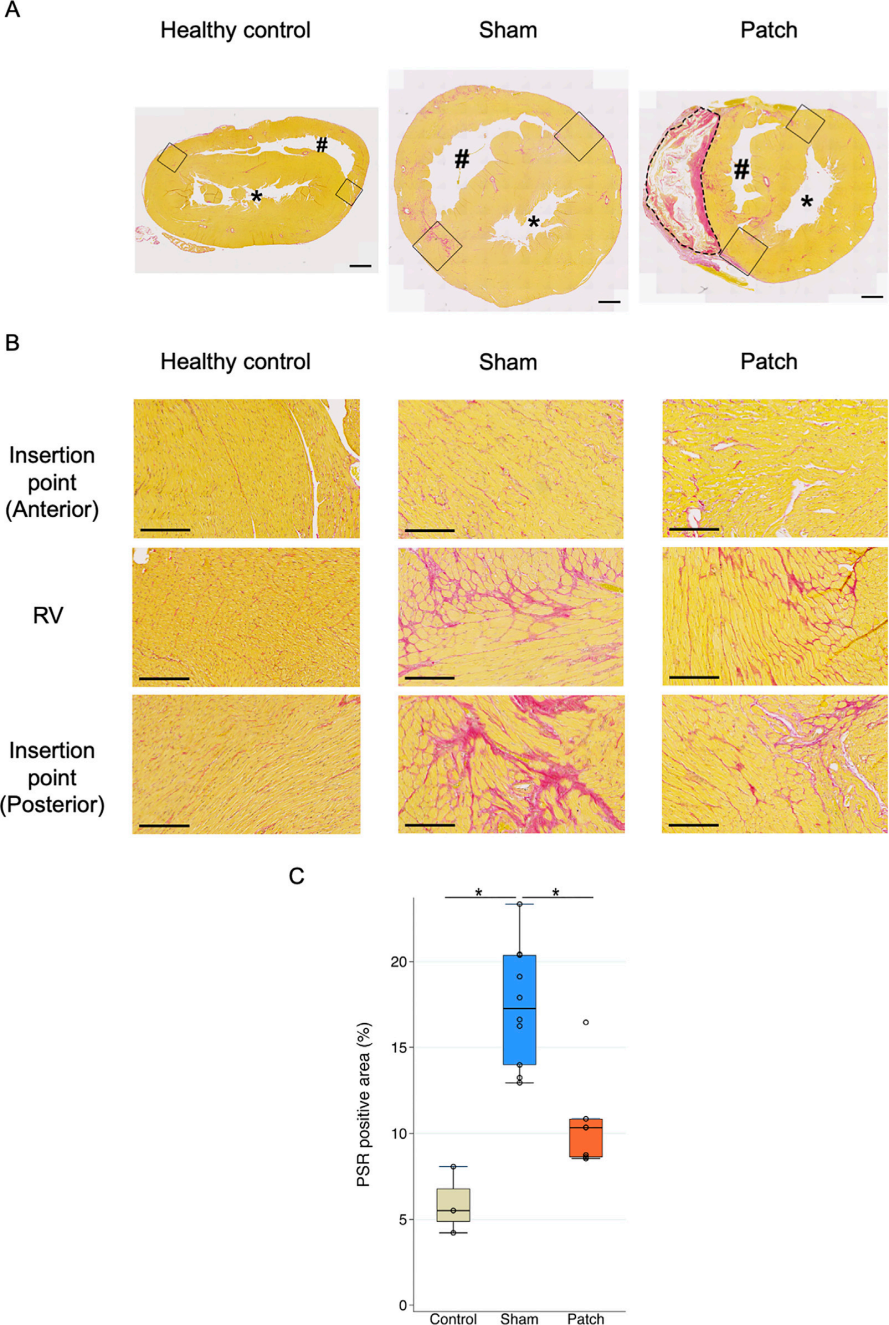
**(A)** Picrosirius red staining. PSR staining showed the accumulation of PSR-positive collagen in the RV and septal insertion point (IP) region in rat hearts 9 weeks after pulmonary artery banding procedures. Scale bars = 1 mm #, right ventricular cavity; *, left ventricular cavity. The remnant patch is enclosed by the dotted line. The right ventricular insertion point is indicated by a black square. **(B)** High magnification of PSR staining showing more accumulation of PSR-positive collagen and myocyte hypertrophy in the sham group although the patch group has a PSR-positive area. Scale bars = 200 um. **(C)** PSR-positive staining to measure fibrotic area in the right ventricle (%). The sham group had more positive area than the other two groups. *p < 0.05.

Pathological interstitial fibrosis 9 weeks after PAB was assessed by PSR staining (Figures 5A, B). The fibrotic area (PSR-positive area) from the RV wall to the RV insertion point increased in the sham and patch groups. In the sham group, more fibrosis was observed in the RV wall and RV insertion point (healthy 5.5 [4.5–7.4], sham 17.3 [quartile range 13.8–20.4], and patch 10.3 [8.6–10.8]; *p* < 0.001, Figure 5C).

### 3.3 Cardiac function

**FIGURE 6:**
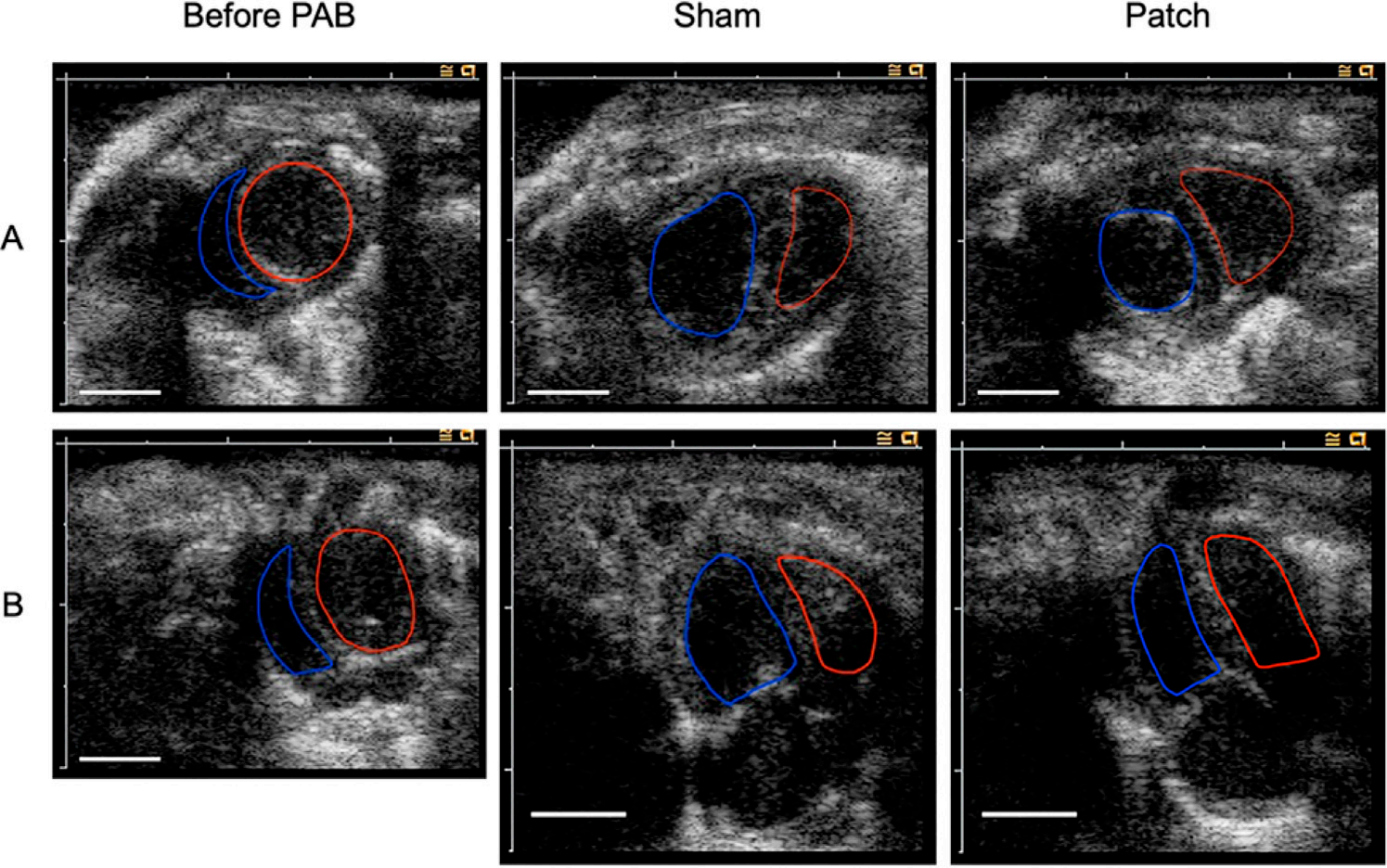
Echocardiography image before PAB and at 9 weeks after for PAB: the sham and patch groups. **(A)** Short-axis view. **(B)** Four chamber view. Red line edges, left ventricular cavity; blue line edges, right ventricular cavity. Scale bars = 5 mm.

The echocardiography images showed right ventricular dilatation and distension of the ventricular septum 9 weeks after PAB (Figure 6). An enlarged RV cavity with a qualitatively diminished left ventricular cavity compared to that before PAB was also apparent.

**FIGURE 7:**
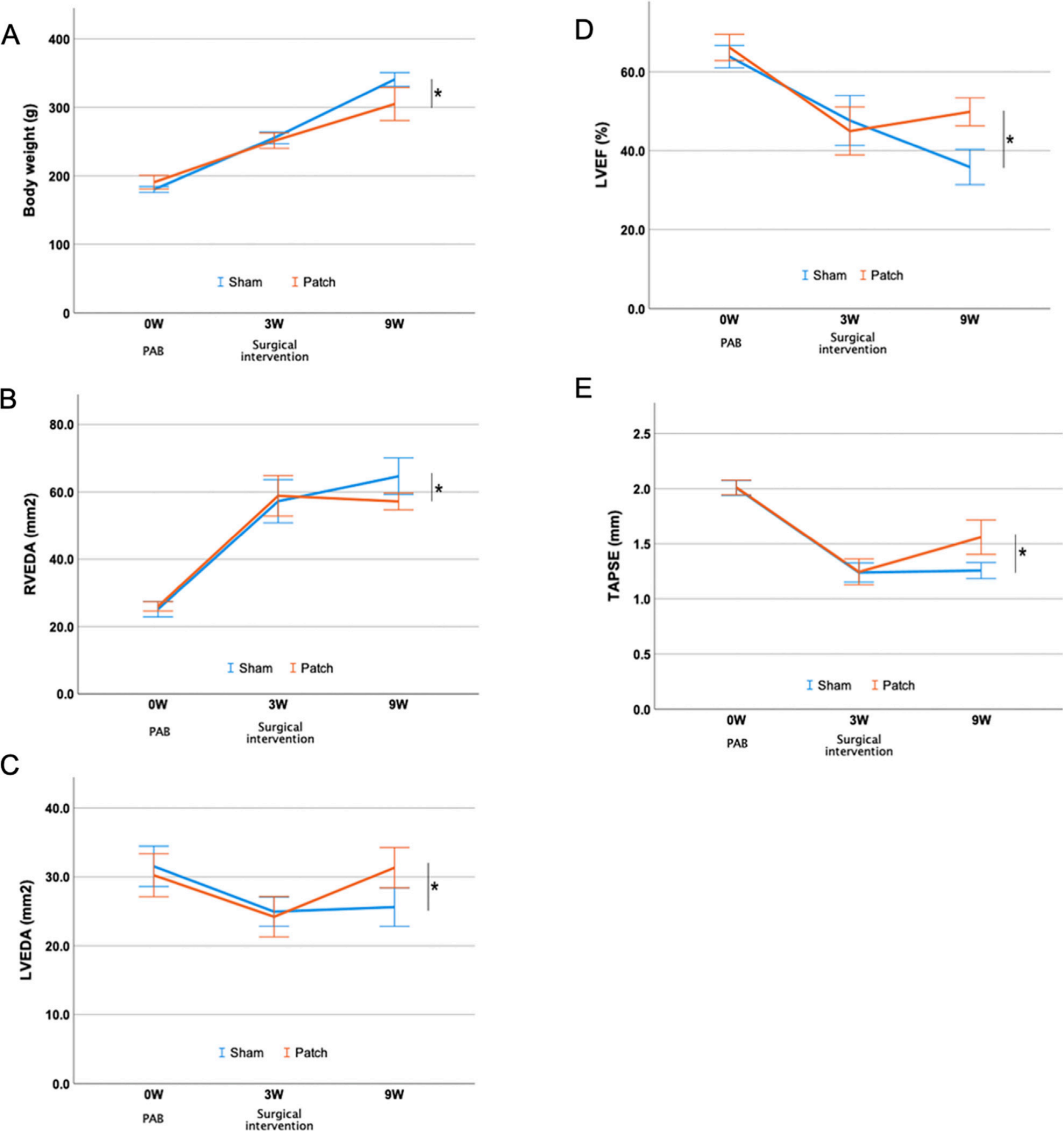
Echocardiography evaluation after PAB and intervention. **(A)** Body weight. **(B**–**E)** Serial changes in right and left ventricular parameters over the follow-up period. *p < 0.05.

The results of echocardiography before surgery and at 3 and 9 weeks after PAB are shown in Figure 7 and Table 1. There were no differences in all parameters of cardiac function between the groups 3 weeks after PAB and before the patch was applied to the patch group. The body weight at 9 weeks after PAB in the sham group was higher than that of the patch group (340.8 ± 5.0 g vs 304.9 ± 12.0 g, *p* = 0.024, Figure 7A). Echocardiography at this time point showed that the RVEDA in the patch group was less dilated than in the sham group (64.7 ± 2.7 vs. 57.2 ± 1.2, *p* = 0.033, Figure 7B). In contrast, LVEDA and LVEF in the patch group were higher than those in the sham group (LVEDA, 25.6 ± 1.4 vs. 31.3 ± 1.5, *p* = 0.015; LVEF, 35.9 ± 2.2 vs. 49.9 ± 1.8, *p* < 0.001, Figures 7C, D). Additionally, TAPSE was increased in the patch vs. sham group at 9 weeks (1.3 ± 0.04 vs. 1.7 ± 0.07, *p* = 0.007, Figure 7E). The proximal and distal RVOT in the patch group was smaller than in the sham group (Table 1).

**TABLE 1:** Echocardiography results 9 weeks after pulmonary artery banding.

	Sham	Patch
LV function		
End-diastolic area	25.6 ± 1.4	31.3 ± 1.5 *
End-systolic area	13.6 ± 0.7	15.2 ± 1.0
% Fractional area change	48.7 [44.6–50.8]	52.2 [51.7–54.0] *
End-diastolic dimension	7.7 ± 0.2	7.8 ± 0.1
End-systolic dimension	6.3 ± 0.2	5.8 ± 0.2
% Fractional shortening	18.0 ± 1.3	26.3 ± 1.1 *
End-diastolic volume	319 ± 22	328 ± 14
End-systolic volume	205.5 ± 17.5	164.7 ± 10.5
Ejection fraction	35.9 ± 2.2	49.9 ± 1.8 *
RV function		
Proximal RV outflow tract	3.3 ± 0.1	2.9 ± 0.1 *
Distal RV outflow tract	2.4 ± 0.1	2.0 ± 0.1 *
End-diastolic area	64.7 ± 2.7	57.2 ± 1.2 *
End-systolic area	45.0 ± 2.9	37.9 ± 1.2
% Fractional area change	30.9 ± 2.3	33.5 ± 2.4
TAPSE	1.26 ± 0.04	1.56 ± 0.08 *

Values are expressed as the mean ± SE or median [interquartile range]. *: *p* < .05 versus the control group.

LV, left ventricle; RV, right ventricle; TAPSE, tricuspid annular plane systolic excursion.

### 3.4 Catheterization data

The results of catheterization 9 weeks after PAB showed that the RV in the patch group had improved pressure loading. Right end-systolic pressure in the patch group was lower than that in the sham group (76.2 ± 5.9 vs 58.2 ± 4.6, *p* = 0.045), and end-diastolic pressure had no differences between the groups. The analysis for the PV loop showed that the end-diastolic pressure–volume relationship in the sham group was higher than in the patch group (1.9 ± 0.2 vs. 1.0 ± 0.4, *p* = 0.043) although there were no significant differences in preload recruitable stroke work and end-systolic pressure–volume relationship (Table 2). Left-sided catheterization showed differences in the left ventricular ejection fraction between the groups.

**TABLE 2:** Catheterizations 9 weeks after pulmonary artery banding.

	Sham	Patch
Left-side catheterization		
Basic hemodynamic index		
Heart rate	218 ± 10	247 ± 13
End-systolic pressure	84 ± 5	68 ± 11
End-diastolic pressure	17.5 ± 2.8	17.2 ± 0.6
Ejection fraction	19 ± 6	30 ± 6 *
Ea	0.71 ± 0.17	0.40 ± 0.11
dP/dt max	1,611 [1,477–3,044]	3,487 [2,659–3,635]
dP/dt min	−2081 ± 516	−1779 ± 328
Tau	54.9 ± 11.5	49.6 ± 10.9
Right-side catheterization		
Basic hemodynamic index		
Heart rate	219 ± 11	228 ± 6
End-systolic pressure	76.2 ± 5.9	58.2 ± 4.6 *
End-diastolic pressure	14.2 ± 1.0	17.2 ± 0.9
Ea	0.61 [0.41–1.06]	0.44 [0.40–0.55]
dP/dt max	1962 ± 244	1,576 ± 205
dP/dt min	−1,651 ± 299	−848 ± 113
Tau	36.0 ± 3.4	50.6 ± 7.0
Load-independent parameters analyzed by the pressure–volume loop		
Preload recruitable stroke work	33.3 ± 5.2	30.5 ± 1.1
End-systolic pressure–volume relationship	445 ± 98	533 ± 81
End-diastolic pressure–volume relationship	1.9 ± 0.2	1.0 ± 0.3 *

Values are expressed as the mean ± SE or median [interquartile range]. *: *p* < 0.05 versus the control group.

### 3.5 Immunofluorescence

**FIGURE 8:**
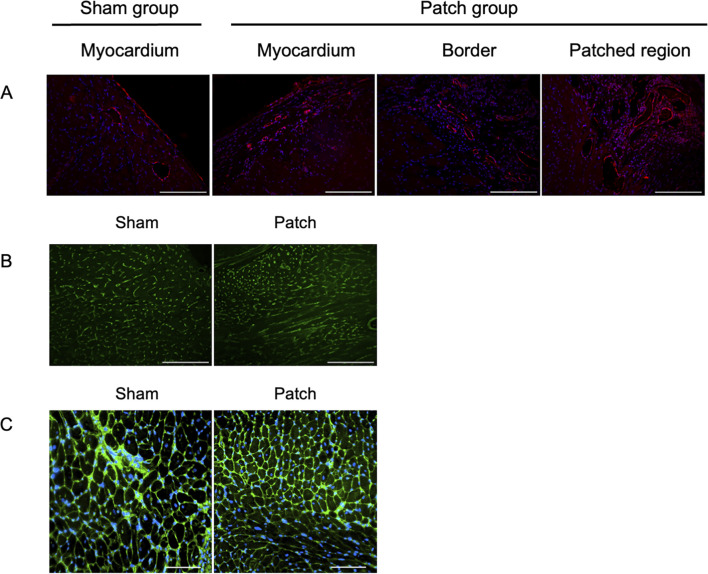
Fluorescence micrographs from the sham and patch groups at 9 weeks after PAB for **(A)** von Willebrand factor (vWF, red) and nuclear (blue) staining, scale bars = 100 um. **(B)** Isolectin staining, scale bars = 100 um, and **(C)** wheat germ agglutinin staining, scale bars = 100 um. For the sham group, the myocardium sample was taken from the location where the patch would have been placed. For the patch group, the myocardium sample was taken from a region distal to the border region.

**FIGURE 9:**
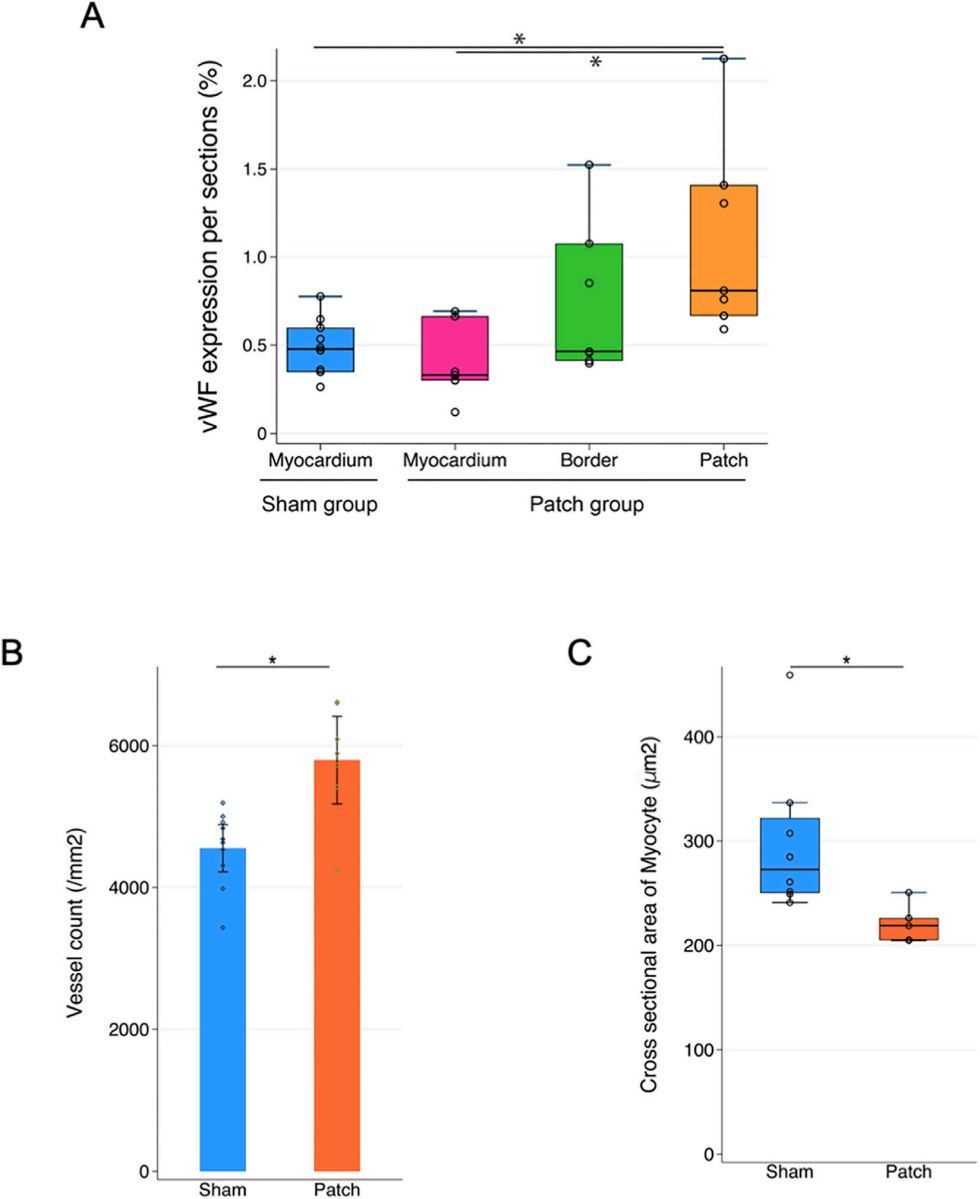
Quantification of immunofluorescence and comparison of **(A)** vWF expression, **(B)** lectin expression, and **(C)** cardiomyocyte size. *p < 0.05.

Vascular density was assessed by immunofluorescence for vWF at 9 weeks post-banding. The density of vWF-staining attributed to vessels was higher in the patch group than that in the sham group (0.81 [0.67–1.41] vs. 0.48 [0.33–0.61], *p* = 0.007, Figures 8A, 9A). In addition, the Isolectin-labeled vessel number in the patch group was higher than that in the sham group (sham vs. patch; 5,794 ± 309 vs 4,551 ± 166, *p* = 0.02, Figures 8B, 9B). Wheat germ agglutinin showed an increased cell size for cardiomyocytes in the sham vs. patch group (273 [250–329] vs. 218 [205–238], *p* = 0.006, Figures 8C, 9C).

### 3.6 Cellular infiltration into the patch

**FIGURE 10:**
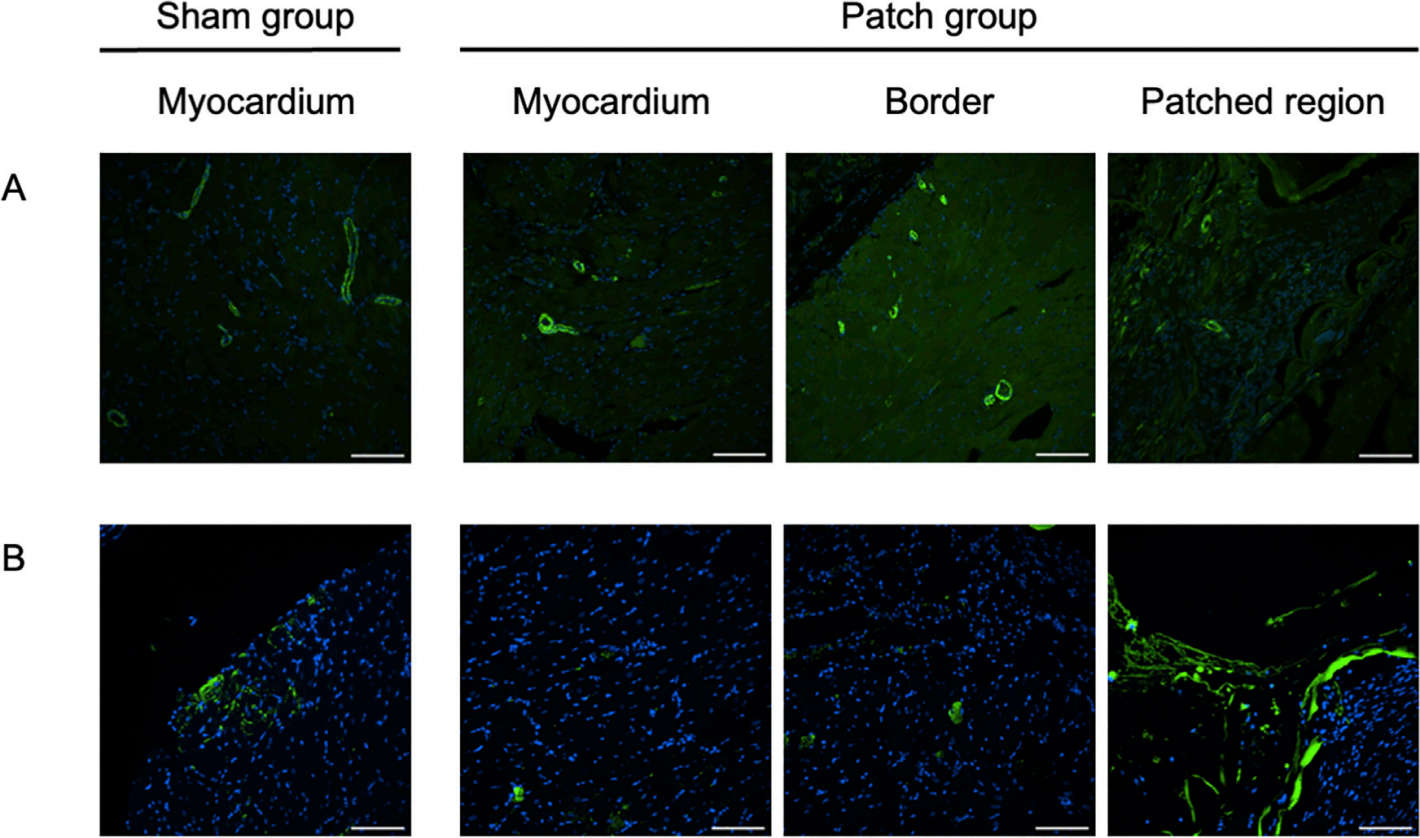
Immunofluorescence staining for **(A)** α-SMA, scale bars = 100 um, and **(B)** CD68, scale bars = 100 um. For the sham group, the myocardium sample was taken from the location where the patch would have been placed. For the patch group, the myocardium sample was taken from a region distal to the border region.

**FIGURE 11:**
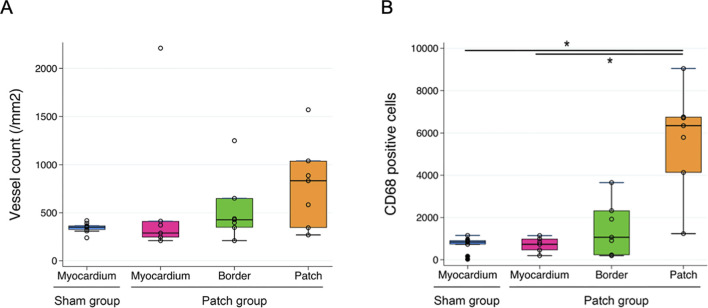
Quantification of immunofluorescence and comparison of **(A)** a-SMA-positive area and **(B)** CD68-positive cells in the myocardium regions *p < 0.05.

The patch applied to the RV wall had obvious cellular infiltration. Vascular density and cellular infiltration were assessed by immunofluorescence for αSMA and CD68 at 9 weeks post-banding. There was no significant difference in the density of αSMA-staining attributed to vessels in the patch between the patch and sham groups (349 [320–372] vs. 834 [345–1,040], *p* = 0.13, Figures 10A, 11A). In addition, CD68-staining showed that the cellular infiltration in the patch group was higher than in the right ventricular wall region that would have been patched in the sham group (sham vs. patch; 834 [588–925] vs. 6,346 [4,132–6,759], *p* < 0.001, Figures 10B, 11B).

## 4 Discussion

There are many studies of right ventricular failure secondary to pulmonary hypertension induced by SU5416 or monocrotaline as models of non-fixed afterload right ventricular failure (Schmuck et al., 2019). However, there are few studies to evaluate epicardial patch placement for right ventricular failure using a fixed-afterload model in which pulmonary artery stenosis is created (Araki et al., 2021; Hoashi et al., 2009). We used the PAB method reported by Hirata et al., which created a consistent degree of pulmonary artery stenosis (Hirata et al., 2015; Andersen et al., 2018). Echocardiography 3 weeks after PAB showed clear evidence of right ventricular hypertrophy and right heart failure (Andersen et al., 2018; Mendes-Ferreira et al., 2016). Histology in the sham group at 9 weeks showed fibrosis of the right ventricle and septal insertion points compared to the patch group, which is comparable to the results with PAB reported by Sun et al. Furthermore, fibrosis was associated with altered right ventricle and pulmonary hemodynamics (Sun et al., 2018; Borgdorff et al., 2012; Andersen et al., 2018).

Our study showed that the biohybrid patch impacted right ventricular wall thickening, cardiomyocyte hypertrophy, and fibrosis and was associated with local angiogenesis. Echocardiographic and catheterization results indicate that patch placement improved diastolic function and right heart failure due to right ventricular overload.

There are reports that mechanical patches prevent cardiac hypertrophy. Currently, a variety of patches have been investigated for myocardial infarction treatment and left heart failure. These include hydrogel patches, fibrous films, elastomeric patches, cell sheets, and microneedle patches (Liu et al., 2024; Domenech et al., 2016). Although some patches rely predominantly on their inherent mechanical or conductive properties to provide cardioprotective effects, many studies have used patches as a platform for loading and delivering cells and other bioactive compounds, including cellular derivatives such as growth factors and exosomes, genes, drugs, and gaseous signaling molecules, which, in turn, may facilitate cardiac recovery or repair.

In an earlier study, the benefit of a cardiac patch design that combines both ventricular mechanical support through a biodegradable, fibrillar elastomeric component and the incorporation of ECM-based hydrogel components was demonstrated (D’Amore et al., 2016). ECM patch implantation on the left ventricle after myocardial infarction revealed favorable remodeling and improved cardiac function, which included decreased left ventricular global mechanical compliance, reduced functional deterioration as measured by echocardiography, less scar formation and left ventricular wall thinning, and increased angiogenesis. This biohybrid patch approach was considered advantageous for application to the PAB right heart failure model, where the mechanical and biological situation is different but where mechanical support might alter the right ventricular remodeling and function in a positive manner.

The initial changes in the right ventricle due to pressure overload are compensatory mechanisms such as volume enlargement and hypertrophic changes to maintain the right ventricular function in an overactive state, and coronary blood flow reserve is maintained at this stage (Reddy and Bernstein, 2015a; Frangogiannis, 2017; Andersen et al., 2019). However, with prolonged pressure loading, the sustained mechanical stress on the myocardium and severe ischemia lead to the metabolic remodeling of cardiomyocytes (Reddy and Bernstein, 2015b). Right ventricular dysfunction progresses to the non-compensatory phase due to reduced cardiac reserve. The use of a biohybrid, elastic, ECM-containing patch for the right ventricle might counteract these processes by providing mechanical support, promoting angiogenesis, and mitigating the inflammatory response.

The data from the current study indicate that patch placement putatively acted to protect the RV from the pressure stress, and by reducing this stress, it also reduced inflammation and fibrosis (Araki et al., 2021; Hoashi et al., 2009). In addition, the cardiac ECM component within the patch may have moderated fibrotic inflammation (D’Amore et al., 2016). This is supported by the data showing that the patch group had less right ventricular fibrosis than the sham group. Additionally, the increased vascularization resulting from right heart patch placement may have reduced microcirculatory deterioration due to hypertrophy. Macrophages (CD68^+^) infiltrating the ECM patches were abundant; however, our previous study showed an increased rate of M2 macrophages infiltrating the patch although the number of macrophages infiltrating the patch increased. In the long term, more M2 infiltrating the ECM patch might suppress immune responses, lead to the production of anti-inflammatory cytokines, and promote angiogenesis. Consistent with this, vWF expression was significantly greater in the patch group, which may also be related to improved myocardial perfusion and the protection of the right ventricular wall.

The biohybrid patch may provide mechanical support to the right ventricle that reduces the mechanical driving force for hypertrophy. The neovascularization that results from patch placement may also reduce ischemia of the right ventricular wall. Both of these effects are considered important, and further experimentation could be carried out with this model to isolate the benefits of a pure neovascularization approach. The biohybrid patch may be considered for use in conditions presenting with right heart failure due to pressure overload, such as pulmonary hypertension, congenital heart disease, and severe left heart failure. In these diseases, the implantation of the patch onto the right ventricle during the early to compensatory phase of right heart failure with pulmonary hypertension may introduce mechanical support and promote angiogenesis, preventing myocardial hypertrophy and myocardial ischemia and progression to the non-compensatory phase.

The ideal patient population for the initial testing of the patch method would be patients with congenital heart disease following surgery. Surgical therapy has improved the prognosis of these patients, but many patients develop right heart failure due to prolonged overload after surgery (Bejleri et al., 2018). The application of this patch technique to a group of patients with congenital heart disease at risk of developing RV failure in post-op, such as Fallot tetralogy and switch (Jatene) operations, may have the potential to improve prognosis.

Limitations in the translation of these findings to larger animals, different intervention time points, or clinical scenarios should be noted, as well as the fact that only one type of patch intervention was studied in this work. In addition, it should be noted that macrophage phenotypes were not pursued. Future studies could evaluate the effect of individual patch parameters, such as ECM content, mechanical properties, and degradation rate. It will also be important to assess cardiomyocyte viability and apoptosis to evaluate the efficacy of the ECM patch. The durability of the observed effects is also of critical importance, particularly after the patch has fully degraded.

## 5 Conclusion

The placement of a biohybrid patch on the right ventricle in a fixed afterload failure model improved hemodynamics and ventricular morphology while increasing regional angiogenesis and reducing right ventricular fibrosis in a rat model. This approach may offer an intervention in scenarios of pulmonary hypertension-driven right heart failure.

## Data Availability

The raw data supporting the conclusions of this article will be made available by the authors, without undue reservation.

## References

[B1] Andersen S. Nielsen-Kudsk J. E. Noordegraaf A. V. de Man F. S. (2019). Right ventricular fibrosis. Circulation 139 (2), 269–285. 10.1161/circulationaha.118.035326 30615500

[B2] Andersen S. Schultz J. G. Holmboe S. Axelsen J. B. Hansen M. S. Lyhne M. D. (2018). A pulmonary trunk banding model of pressure overload induced right ventricular hypertrophy and failure. J. Vis. Exp. JoVE 141 (November). 10.3791/58050 30582605

[B3] Araki K. Miyagawa S. Kawamura T. Ishii R. Watabe T. Harada A. (2021). Autologous skeletal myoblast patch implantation prevents the deterioration of myocardial ischemia and right heart dysfunction in a pressure-overloaded right heart porcine model. PloS One 16 (2), e0247381. 10.1371/journal.pone.0247381 33635873 PMC7909703

[B4] Bejleri D. Streeter B. W. Nachlas A. L. Y. Brown M. E. Gaetani R. Christman K. L. (2018). A bioprinted cardiac patch composed of cardiac-specific extracellular matrix and progenitor cells for heart repair. Adv. Healthc. Mater. 7 (23), e1800672. 10.1002/adhm.201800672 30379414 PMC6521871

[B5] Borgdorff M. A. J. Bartelds B. Dickinson M. G. Boersma B. Weij M. Zandvoort A. (2012). Sildenafil enhances systolic adaptation, but does not prevent diastolic dysfunction, in the pressure-loaded right ventricle. Eur. J. Heart Fail. 14 (9), 1067–1074. 10.1093/eurjhf/hfs094 22730335

[B6] D’Amore A. Yoshizumi T. Luketich S. K. Wolf M. T. Gu X. Cammarata M. (2016). Bi-layered polyurethane - extracellular matrix cardiac patch improves ischemic ventricular wall remodeling in a rat model. Biomaterials 107 (November), 1–14. 10.1016/j.biomaterials.2016.07.039 27579776

[B7] Domenech M. Polo-Corrales L. Ramirez-Vick J. E. Freytes D. O. (2016). Tissue engineering strategies for myocardial regeneration: acellular versus cellular scaffolds? Tissue Eng. Part B, Rev. 22 (6), 438–458. 10.1089/ten.teb.2015.0523 27269388 PMC5124749

[B8] Frangogiannis N. G. (2017). Fibroblasts and the extracellular matrix in right ventricular disease. Cardiovasc. Res. 113 (12), 1453–1464. 10.1093/cvr/cvx146 28957531 PMC5852546

[B9] Hashizume R. Hong Y. Takanari K. Fujimoto K. L. Tobita K. Wagner W. R. (2013). The effect of polymer degradation time on functional outcomes of temporary elastic patch support in ischemic cardiomyopathy. Biomaterials 34 (30), 7353–7363. 10.1016/j.biomaterials.2013.06.020 23827185 PMC3804157

[B10] Hirata M. Ousaka D. Arai S. Okuyama M. Tarui S. Kobayashi J. (2015). Novel model of pulmonary artery banding leading to right heart failure in rats. BioMed Res. Int. 2015 (October), 1–10. 10.1155/2015/753210 PMC4609378 26504827

[B11] Hoashi T. Matsumiya G. Miyagawa S. Ichikawa H. Ueno T. Ono M. (2009). Skeletal myoblast sheet transplantation improves the diastolic function of a pressure-overloaded right heart. J. Thorac. Cardiovasc. Surg. 138 (2), 460–467. 10.1016/j.jtcvs.2009.02.018 19619796

[B12] Lang R. M. Badano L. P. Mor-Avi V. Afilalo J. Armstrong A. Ernande L. (2015). Recommendations for cardiac chamber quantification by echocardiography in adults: an update from the American society of echocardiography and the European association of cardiovascular imaging. Eur. Heart J. Cardiovasc. Imaging 16 (3), 233–271. 10.1093/ehjci/jev014 25712077

[B13] Liu T. Ying H. Zhang Z. Zhou H. Peng S. Zhang D. (2024). Advanced cardiac patches for the treatment of myocardial infarction. Circulation 149 (25), 2002–2020. 10.1161/circulationaha.123.067097 38885303 PMC11191561

[B14] Ma Z. Mao L. Rajagopal S. (2016). Hemodynamic characterization of rodent models of pulmonary arterial hypertension. J. Vis. Exp. JoVE 110 (April), 53335. 10.3791/53335 PMC4941906 27167679

[B15] Mendes-Ferreira P. Santos-Ribeiro D. Adão R. Maia-Rocha C. Mendes-Ferreira M. Sousa-Mendes C. (2016). Distinct right ventricle remodeling in response to pressure overload in the rat. Am. J. Physiology. Heart Circulatory Physiology 311 (1), H85–H95. 10.1152/ajpheart.00089.2016 27199115

[B16] Meyer P. Filippatos G. S. Ahmed M. I. Iskandrian A. E. Bittner V. Perry G. J. (2010). Effects of right ventricular ejection fraction on outcomes in chronic systolic heart failure. Circulation 121 (2), 252–258. 10.1161/circulationaha.109.887570 20048206 PMC2877272

[B17] Reddy S. Bernstein D. (2015a). The vulnerable right ventricle. Curr. Opin. Pediatr. 27 (5), 563–568. 10.1097/mop.0000000000000268 26262580 PMC7441820

[B18] Reddy S. Bernstein D. (2015b). Molecular mechanisms of right ventricular failure. Circulation 132 (18), 1734–1742. 10.1161/circulationaha.114.012975 26527692 PMC4635965

[B19] Remlinger N. T. Wearden P. D. Gilbert T. W. (2012). Procedure for decellularization of porcine heart by retrograde coronary perfusion. J. Vis. Exp. JoVE 70 (December), e50059. 10.3791/50059 PMC3567168 23242494

[B20] Schmuck E. G. Hacker T. A. Schreier D. A. Chesler N. C. Wang Z. (2019). Beneficial effects of mesenchymal stem cell delivery via a novel cardiac bioscaffold on right ventricles of pulmonary arterial hypertensive rats. Am. J. Physiology. Heart Circulatory Physiology 316 (5), H1005–H1013. 10.1152/ajpheart.00091.2018 PMC6580387 30822119

[B21] Sun M. Ishii R. Okumura K. Krauszman A. Breitling S. Gomez O. (2018). Experimental right ventricular hypertension induces regional β1-integrin–mediated transduction of hypertrophic and profibrotic right and left ventricular signaling. J. Am. Heart Assoc. 7 (7), e007928. 10.1161/JAHA.117.007928 29599211 PMC5907585

[B22] Trac D. Maxwell J. T. Brown M. E. Xu C. Davis M. E. (2019). Aggregation of child cardiac progenitor cells into spheres activates notch signaling and improves treatment of right ventricular heart failure. Circulation Res. 124 (4), 526–538. 10.1161/circresaha.118.313845 30590978 PMC6375764

[B23] Yogeswaran A. Mamazhakypov A. Schermuly R. T. Weiß A. (2023). Right ventricular failure in pulmonary hypertension: recent insights from experimental models. Herz 48, 285–290. 10.1007/s00059-023-05180-8 37079028

